# Galectina-3 Associada a Formas Graves e Mortalidade em Longo Prazo em Pacientes com Doença de Chagas

**DOI:** 10.36660/abc.20190403

**Published:** 2021-02-19

**Authors:** Fábio Fernandes, Carlos Henrique Valente Moreira, Lea Campos Oliveira, Marcela Souza-Basqueira, Barbara Maria Ianni, Claudia di Lorenzo, Felix José Alvarez Ramires, Luciano Nastari, Edecio Cunha-Neto, Antonio L. Ribeiro, Renato Delascio Lopes, Sheila M. Keating, Ester Cerdeira Sabino, Charles Mady

**Affiliations:** 1 Universidade de São PauloFaculdade de MedicinaHospital das ClínicasSão PauloSPBrasilUniversidade de São Paulo Faculdade de Medicina Hospital das Clínicas Instituto do Coração, São Paulo, SP- Brasil; 2 Instituto de Infectologia Emilio RibasSão PauloSPBrasilInstituto de Infectologia Emilio Ribas, São Paulo, SP - Brasil; 3 Universidade de São PauloFaculdade de MedicinaSão PauloSPBrasilUniversidade de São Paulo Faculdade de Medicina, São Paulo, SP - Brasil; 4 Universidade Federal de Minas GeraisCentro de TelessaúdeHospital das ClínicasBelo HorizonteMGBrasilUniversidade Federal de Minas Gerais - Centro de Telessaúde - Hospital das Clínicas, Belo Horizonte, MG - Brasil; 5 Duke University HospitalDurhamNorth CarolinaEUADuke University Hospital, Durham, North Carolina - EUA; 6 Blood Systems Research InstituteSan FranciscoCalifórniaEUABlood Systems Research Institute, San Francisco, Califórnia – EUA

**Keywords:** Doença de Chagas, Cardiomiopatia Chagásica, Mortalidade, Galectina 3, Biomarcadores, Eletrocardiografia/métodos, Insuficiência Cardíaca

## Abstract

**Fundamento:**

As características histopatológicas da doença de Chagas (DCC) são: presença de miocardite, destruição das fibras cardíacas e fibrose miocárdica. A Galectina-3 (Gal-3) é um biomarcador envolvido no mecanismo de fibrose e inflamação que pode ser útil para a estratificação de indivíduos com DCC por risco.

**Objetivos:**

Nosso objetivo foi avaliar se níveis elevados de Gal-3 estão associados a formas graves de cardiomiopatia chagásica (CC) e são preditivos de mortalidade.

**Métodos:**

Estudamos doadores de sangue (DS) positivos para anti-*T. cruzi*: não-CC-DS (187 DS sem CC com eletrocardiograma [ECG] e fração de ejeção do ventrículo esquerdo [FEVE] normais); CC-Não-Dis-DS (46 DS com CC e apresentando ECG anormal, mas FEVE normal); e 153 controles negativos correspondentes. Esta amostra foi composta por 97 pacientes com CC grave (CC-Dis). Usamos as correlações de Kruskall-Wallis e Spearman para testar a hipótese de associações, assumindo um p bicaudal <0,05 como significativo.

**Resultados:**

O nível de Gal-3 foi de 12,3 ng/mL para não-CC-DS, 12,0 ng/mL para CC-Não-Dis-DS, 13,8 ng/mL para controles e 15,4 ng/mL para CC-Dis. FEVE <50 foi associada a níveis mais elevados de Gal-3 (p=0,0001). Em nosso modelo de regressão linear ajustado, encontramos associação entre os níveis de Gal-3 e os parâmetros do ecocardiograma em indivíduos positivos para *T. cruzi*. Nos pacientes CC-Dis, encontramos uma associação significativa de níveis mais elevados de Gal-3 (≥15,3 ng/mL) e morte ou transplante cardíaco em acompanhamento de cinco anos (Hazard ratio – HR 3,11; IC95% 1,21– 8,04; p=0,019).

**Conclusões:**

Em pacientes com CC, níveis mais elevados de Gal-3 estiveram significativamente associados a formas graves da doença e maior taxa de mortalidade em longo prazo, o que significa que pode ser um meio efetivo para identificar pacientes de alto risco. (Arq Bras Cardiol. 2021; 116(2):248-256)

## Introdução

A cardiomiopatia chagásica (CC), uma das principais causas de cardiopatia e morte na América Latina, tem um prognóstico ruim em comparação às cardiomiopatias não inflamatórias.^1^

O curso natural da doença de Chagas (DCC) envolve uma fase aguda, seguida da fase crônica. Ainda não se sabe, entretanto, quais pacientes têm maior probabilidade de progredir para as formas graves. Lesão direta pelo parasita, inflamação desencadeada pelo sistema imunológico e disfunção autonômica são fatores importantes na patogênese da CC. Quando o tecido cardíaco sofre lesão, a fibrose de substituição parece ser uma causa de desorganização estrutural e geométrica, e prejuízo funcional do coração.^2^

A galectina-3 (Gal-3) é secretada por macrófagos ativados e está envolvida na fibrogênese da insuficiência cardíaca (IC). Tal biomarcador foi recentemente associado ao desenvolvimento de IC e mortalidade. Em um modelo experimental de DCC, a Gal-3 promoveu infiltração celular no coração e fibrose.^3,4^

A falta de um bom marcador de infecção ativa ou CC incipiente torna o desenvolvimento de novos tratamentos nessa população um desafio. O uso de biomarcadores que podem prever com precisão os resultados clínicos na CC teria o potencial de orientar a terapia, identificando pacientes com maior risco e que necessitariam de intervenção mais precoce, intensiva e personalizada.^5^

O objetivo do nosso estudo foi avaliar se níveis elevados de Gal-3 estão associados a formas graves da CC e se trata-se de um fator preditivo de mortalidade ou necessidade de transplante cardíaco.

## Métodos

### Desenho do Estudo

As amostras foram coletadas durante o *Retrovirus Epidemiology Donor Study-II* (REDS-II),^6^ um estudo retrospectivo em que doadores de sangue (DS) soropositivos para *T. cruzi* foram identificados por triagem em bancos de sangue em 1996-2002 (233 da cidade de São Paulo), além de 153 doadores-controle soronegativo (pareados por ano de doação, idade e sexo). A amostra foi composta por 97 casos de CC previamente diagnosticados pelo Instituto do Coração (INCOR) da Faculdade de Medicina, Universidade de São Paulo. De julho de 2008 a outubro de 2010, indivíduos recrutados (pacientes DS e com CC) preencheram questionários de saúde e passaram por avaliações médicas, incluindo eletrocardiograma (ECG), ecocardiograma (ECO) e flebotomia com processamento e criopreservação de amostras para subsequentes análises cegas em lote de marcadores cardíacos, reação em cadeia da polimerase (PCR) para detecção de *T. cruzi* e outros biomarcadores (ver abaixo).

Primeiro, realizamos um estudo transversal em que participantes positivos para *T. cruzi* preencheram um questionário e passaram por uma avaliação médica (parâmetros laboratoriais, ECG e ECO), resultando em grupos estratificados pelo status da CC. Em seguida, foi realizado um estudo de coorte longitudinal, no qual 97 pacientes soropositivos para *T. cruzi*, portadores de formas crônicas e mais graves de CC, foram acompanhados em um serviço ambulatorial do INCOR, onde havia dados sobre “tempo até o evento”.

Todas as amostras de sangue foram coletadas em tubos de EDTA e de soro, processadas para detecção do parasita ou submetidas à centrifugação e divididas em alíquotas. Todas as amostras foram congeladas no Brasil a -20 °C até o envio ao Laboratório Central REDS-II (*Blood Systems Research Institute*, San Francisco, CA) em gelo seco e mantidas a -70 °C.

Foram criados quatro grupos: um grupo controle soronegativo e três grupos soropositivos para *T. cruzi*: DS sem cardiomiopatia, apresentando ECG normal e fração de ejeção ventricular esquerda (FEVE) (Não-CC-DS); DS com CC e apresentando anormalidades no ECG, mas função sistólica normal (CC-Não-Dis-DS); e participantes com CC e disfunção ventricular esquerda (CC-Dis). A disfunção foi definida como FEVE <50% identificada no ECO.

Os dados relativos à análise de tempo até o evento estavam disponíveis apenas para os indivíduos do grupo CC-Dis. De julho a setembro de 2015, procedemos com a análise do prontuário e ligações para os pacientes com CC-Dis para monitorar eventos e anotar as respectivas datas.

Os comitês de ética locais aprovaram o protocolo do estudo e todos os participantes assinaram o termo de consentimento livre e esclarecido.

### Procedimentos PCR

Coletamos de cada participante 20 mL de sangue anticoagulado com EDTA que foram imediatamente misturados com igual volume de solução de cloridrato de guanidina 6M/EDTA -0,2 M. O teste de PCR em tempo real de captura de alvo utilizado neste estudo foi desenvolvido com base no método PCR descrito por Virreira et al.,^7^ visando o DNA do minicírculo mitocondrial do cinetoplasto *T. cruzi*. A extração do DNA foi aprimorada com uma etapa de captura do alvo usando esferas magnéticas revestidas com um oligonucleotídeo de captura 20-mer específico para *T. cruzi*.

### Medidas Cardíacas

Foram registrados ECGs de repouso de 12 derivações (eletrocardiógrafo General Electric MAC 1200; GE Healthcare, Waukesha, WI).^8,9^

Os ecocardiogramas foram realizados em aparelho de ultrassom Sequoia^TM^ 512 (Acuson, Mountain View, CA), de acordo com as diretrizes da American Society of Echocardiography.^10,11^ Os estudos foram gravados em formato digital e todas as medidas foram feitas em loops digitais, em uma estação de análise offline Digisonics (software versão 3.2; Digisonics, Houston, TX) no laboratório de ecocardiografia do departamento cardiovascular, no *National Heart, Lung, and Blood Institute* (Bethesda, MD).^12^

### Biomarcadores Solúveis

Biomarcadores associados à infecção por *T. cruzi* foram descritos anteriormente.^12^ Amostras de plasma foram testadas usando kits Milliplex (Millipore) com grânulos revestidos de anticorpo para detecção do fator de necrose tumoral alfa (TNF-α), interleucina (IL) 6 (IL-6), IL-8, IL-10 e interferon gama (IFNγ). Curvas e amostras-padrão foram testadas em duplicata. Os resultados foram adquiridos em um analisador Labscan 200 (Luminex) usando o software gerenciador Bio-Plex v6.1 (Bio-Rad). O IFN- esteve, na maioria dos casos, abaixo do limiar de detecção (57%).

As concentrações de peptídeo natriurético do tipo B (NT-proBNP) e troponina foram medidas em testes aprovados pela Food and Drug Administration (FDA) no Sistema VITROS (Ortho Clinical Diagnostics, Raritan, NJ).

Os níveis plasmáticos de Gal-3 foram determinados por um teste de fluorescência ligada à enzima e medidos em um sistema BioMerieux Vidas 30 (BioMerieux, Marcy l’Etoile, Lyon, França), seguindo as recomendações do fabricante.

### Análise Estatística

A normalidade foi avaliada pelo teste de Shapiro-Wilk. As variáveis contínuas sem distribuição normal foram: idade, níveis de Gal-3, fração de ejeção e outros biomarcadores cardíacos e inflamatórios, todos expressos em mediana e intervalo interquartil. As diferenças entre os grupos quanto às variáveis foram comparadas com o teste de Kruskal-Wallis, enquanto o teste do qui-quadrado ou de Fisher e a regressão logística foram usados para avaliar o tipo e a distribuição das variáveis. As classificações na análise de variância de um critério e análise post-hoc foram feitas por meio do teste de Dunn para avaliar as diferenças de valores medianos entre os grupos com ajuste de Bonferroni para comparações múltiplas. As correlações foram analisadas pela correlação de Spearman, reportando-se valores de p.

Curvas de características de operação do receptor (curva ROC) foram criadas para as variáveis Gal-3 e NT-proBNP para otimizar a definição do ponto de corte que melhor discriminaria o evento durante o acompanhamento, e foi identificada uma área sob a curva ROC (AUC). Ambas foram comparadas pelos testes DeLong e qui-quadrado.

Para a análise de tempo até o evento, o grupo CC-Dis foi dividido em dois perfis em relação aos valores de corte para Gal-3 e NT-proBNP: Gal-3 ou NT-proBNP baixos (≤ valor de corte) e Gal-3 ou NT-proBNP altos (≥ valor de corte). A incidência de eventos cumulativos nos estratos Gal-3 e NT-proBNP e o valor aditivo de Gal-3 em relação ao NT-proBNP foram analisados por um método semelhante a Kaplan-Meier, seguido do teste log-rank.

Modelos de regressão de risco proporcional de Cox bivariados e multivariados foram construídos para avaliar a associação dos valores de Gal-3 e NT-proBNP (abaixo *versus *acima ou igual ao valor de corte) com incidentes e eventos. Os modelos foram ajustados para sexo, idade, creatinina sérica, classificação da New York Heart Association (NYHA) e FEVE; Intervalos de confiança de 95% (IC) foram usados para representar a associação de cada marcador com os eventos no modelo final de risco proporcional de Cox ajustado. A sensibilidade foi analisada usando os valores de Gal-3 e NT-proBNP como variáveis contínuas. Valor de p<0,05 bicaudal foi considerado significativo.

Todos os gráficos e análises estatísticas foram feitos no software Stata (versão 13.0, Stata Corp., College Station, TX).

## Resultados

**Figura 1:**
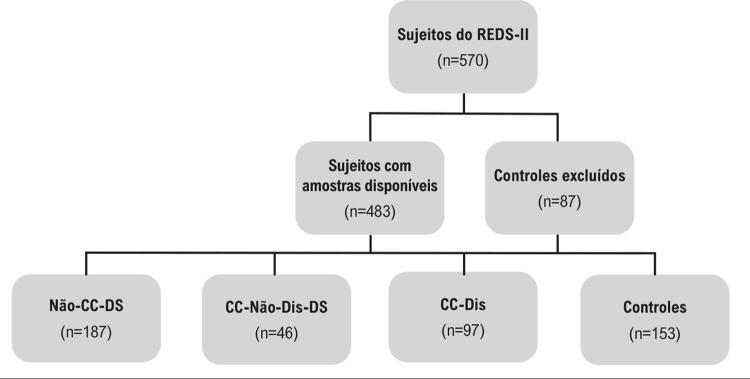
– Fluxograma de inclusão.

Dos 570 participantes originais do REDS-II, 483 tinham amostras disponíveis para o teste Gal-3; 153 eram negativos para *T. cruzi* e os demais foram classificados em três grupos: 187 Não-CC-DS apresentando ECG e FEVE normais; 46 CC-não-Dis; e 97 CC-Dis (Figura 1).

*CC: cardiomiopatia chagásica; Não-CC-DS: doadores de sangue com sorologia positiva para T. cruzi e sem cardiomiopatia, apresentando eletrocardiograma (ECG) e fração de ejeção do ventrículo esquerdo (FEVE) normais; CC-Não-Dis-DS: doadores de sangue com sorologia positiva para T. cruzi apresentando anormalidades no ECG e FEVE normal em repouso; CC-Dis: pacientes com sorologia positiva para T. cruzi e CC, com disfunção ventricular esquerda.*

### Características Clínicas e Biomarcadores dos Pacientes

As características demográficas e clínicas estão descritas na Tabela 1. Os níveis de Gal-3 estavam mais altos nos pacientes do grupo CC-Dis, em comparação com outros grupos clínicos. Níveis mais elevados de Gal-3 também foram observados nos controles quando comparados aos não-CC-DS. Não observamos diferenças significativas nos níveis de Gal-3 entre Não-CC-DS e CC-não-Dis-DS, ou entre CC-não-Dis-DS e controles. Marcadores inflamatórios (TNF-α, IL-6, IL-8, IL-10), bem como biomarcadores associados à disfunção ou dano cardíaco (NT-proBNP e troponina) estavam elevados em pacientes com CC-Dis em comparação aos outros grupos (Tabela 1).

**Tabela 1:** – Achados clínicos, laboratoriais e ecocardiográficos

	Não-CC-DS	CC-não-Dis-DS	CC-Dis	Controles	
	n=187 (38,6%)	n=46 (9,5%)	n=97 (20,2%)	n=153 (31,6%)	valor de p
Idade (anos)	49 [41-58]	50 [44-59]	48,5 [43-54]	48 [42-55]	0,20
Masculino, n (%)	110 (58,8)	29 (63)	59 (60,2)	89 (58,2)	0,39
Fração de ejeção (%)	63 [60-65]	60 [55-65]	30 [20-40]	64 [60-65]	<0,001*
**Biomarcadores cardíacos**					
Galectina-3 (ng/mL)	12,3 [10-15,4]	12,0 [9,5-14,9]	15,4 [11,8-19,8]	13,8 [11,2-16,2]	<0,001^†^
NT-proBNP (pg/mL)	40,6 [23,6-66,8]	59 [35,0-109,1]	748 [379,20-2223,41]	27,5 [19,3-48,1]	<0,001^‡^
Troponina	0,012 [0,01-0,012]	0,012 [0,012-0,015]	0,021 [0,12-0,03]	0,012 [0,012-0,012]	<0,001^§^
**Marcadores inflamatórios**					
TNF-α	2,94 [1,64-4,59]	3,02 [1,25-4,69]	3,65 [2,57-5,52]	2,84 [1,62-3,91]	0,002^#^
IL-6	0,69 [0,32-1,63]	0,77 [0,32-1,8]	1,60 [0,64-3,13]	1,14 [0,32-1,7]	<0,001**
IL-8	1,61 [0,95-2,79]	1,53 [0,99-2,5]	2,23 [1,38-3,2]	1,44 [0,95-2,54]	0,003^††^
IL-10	1,28 [0,32-4]	2,02 [0,32-4,11]	4,37 [1,62-8,06]	1,22 [0,32-3,35]	<0,001^‡‡^
IFN-γ	0,32 [0,32-0,64]	0,32 [0,32-0,84]	0,32 [0,32-1,07]	0,32 [0,32-0,39]	0,06
**Carga parasitária**					
Estimativa do parasita por 20mL	0,05 [0-2,5]	0,68 [0,03-5,47]	1,77 [0,16-5]	-	<0,001^§§^

Valores de p foram reportados para os testes de hipótese post-hoc de Kruskal-Wallis e Dunn.

Mediana [intervalo interquartil] reportada para todos os biomarcadores testados. Não-CC-DS, doadores de sangue sem cardiomiopatia chagásica; CC-Não-Dis-DS, doadores de sangue com cardiomiopatia chagásica; CC-Dis, pacientes com cardiomiopatia chagásica e disfunção cardíaca; NT-proBNP, N-terminal do pró-hormônio do peptídeo natriurético do tipo B; TNF, fator de necrose tumoral; IL, interleucina; IFN, interferon.

* Diferença estatisticamente significativa nos níveis de fração de ejeção entre Não-CC-DS /CC-Dis (p<0.001); CC-Não-Dis-DS/CC-Dis (p<0,001); CC-Não-Dis-DS/controles (p=0,042); CC-Dis/controles (p<0,001).

†Diferença estatisticamente significativa nos níveis de Gal-3 entre Não-CC-DS/CC-Dis (p<0,001); Não-CC-DS/controles (p=0,010); CC-Não-Dis-DS/CC-Dis (p<0,001); CC-Dis/controles (p=0,028).

‡Diferença estatisticamente significativa nos níveis de NT-proBNP entre Não-CC-DS/CC-Dis (p<0,001); Não-CC-DS/controles (p=0.004); CC-Não-Dis-DS/CC-Dis (p<0,001); CC-Não-Dis-DS/controles (p<0,001); CC-Dis/controles (p<0,001).

§ Diferença estatisticamente significativa nos níveis de troponina entre Não-CC-DS/CC-Dis (p=0,024); Não-CC-DS/CC-controles (p<0,001); CC-Não-Dis-DS/CC-Dis (p<0,001); CC-Não-Dis-DS/controles (<0,001); CC-Dis/controles (p<0,001).

#Diferença estatisticamente significativa nos níveis de TNF-α entre Não-CC-DS/CC-Dis (p=0,019); CC-Dis/controles (p<0,001).

**Diferença estatisticamente significativa nos níveis de IL-6 entre Não-CC-DS/CC-Dis (p<0,001); CC-Não-Dis-DS/CC-Dis (p=0,032); CC-Dis/controles (p=0,004).

††Diferença estatisticamente significativa nos níveis de IL-8 entre Não-CC-DS/CC-Dis (p=0,016); CC-Não-Dis-DS/CC-Dis (p=0,039); CC-Dis/controles (p=0,001).a

‡‡ Diferença estatisticamente significativa nos níveis de IL-10 entre Não-CC-DS/CC-Dis (p<0,001); CC-Não-Dis-DS/CC-Dis (p=0,001); CC-Dis/controles (p<0,001).

§§ Diferença estatisticamente significativa na estimativa do parasita por 20mL entre Não-CC-DS/CC-Dis (p=0,011); Não-CC-DS/controles (p<0,001).

A PCR positiva para *T. cruzi* indicou diferença estatisticamente significativa entre os grupos Não-CC-DS e CC: CC-Não-Dis-DS (p=0,010) e CC-Dis. Por outro lado, não foi observada diferença na parasitemia ao comparar os grupos CC-Não-Dis-DS e CC-Dis (Tabela 1). Também não encontramos diferença significativa em pacientes com CC-Dis entre a PCR para *T. cruzi* e a ocorrência do evento. Nenhuma associação significativa entre PCR para *T. cruzi* e Gal-3 foi encontrada.

A correlação de Spearman foi aplicada entre os indivíduos infectados por *T. cruzi*, para avaliar a relação entre Gal-3 e biomarcadores cardíacos, mediadores inflamatórios e carga parasitária. Houve uma correlação fraca para TNF-α (rs=0,25, p<0,001) e IL-8 (rs=0,22, p<0,001). Nenhuma associação entre Gal-3 e troponina, NT-proBNP, IL-6, IL-10, IFNγ, ou com carga parasitária foi observada.

O ecocardiograma foi realizado, correlação de Spearman, para verificar qualquer relação entre os níveis de Gal-3 e parâmetros ecocardiográficos em pacientes infectados por *T. cruzi*. Nenhuma correlação estatisticamente significativa moderada ou forte foi encontrada entre os níveis de Gal-3 e o diâmetro diastólico final do ventrículo esquerdo (DDFVE) (rs=0,09, p=0,07), dimensão sistólica final do ventrículo esquerdo (DSFVE) (rs=0,11, p=0,03), FEVE (rs=-0,16, p=0,001), diâmetro do átrio esquerdo (rs=0,11, p=0,02), volume atrial esquerdo indexado à área de superfície corporal (rs=0,09, p=0,18), área do átrio direito (rs=0,032, p=0,53) e relação E/e’ septal (rs=0,135, p=0,009).

### Sobrevivência e Análise de Risco

Os dados sobre tempo até o evento estavam disponíveis para 97 pacientes, com seguimento médio de 51,2 ± 10,8 meses e mediana de 58 meses (variação: 8 a 60 meses). Foram observados eventos em 28 pacientes (29%): três (10,8%) transplantes cardíacos e 25 (89,2%) óbitos por todas as causas. Entre os pacientes envolvidos em eventos, as concentrações médias de Gal-3 e NT-pro-BNP foram significativamente maiores, enquanto a fração de ejeção foi significativamente menor. Idade, sexo, classe NYHA>I, fração de ejeção na ecocardiografia e dados laboratoriais de pacientes com evento relatado estão na Tabela 2.

**Tabela 2:** – Informações demográficas e laboratoriais dos pacientes com CC-Dis segundo óbito ou transplante cardíaco

	Sem eventos	Eventos	valor de p
	(n=69)	(n=28)	
Idade (anos)	49 [42-54]	47,5 [44,5-52]	0,96
Masculino, n (%)	42 (61%)	16 (57%)	0,73
Creatinina (mg/dL)	1,01 [0,85-1,14]	1,14 [0,87-1,23]	0,06
Fração de ejeção (%)	35 [25-40]	20 [20-30]	0,001
NYHA >1	29	20	0,009
Galectina-3 (ng/mL)	14,4 [10,9-19,1]	18,5 [14,7-23,4]	0,005
Baixo	39	7	
Alto	30	21	
NT-proBNP (pg/mL)	542 [281-1337]	2643 [1047-4771]	<0,001
Baixo	50	7	
Alto	19	21	
Estimativa parasitária*	1,77 [0,19-4,2]	1,25 [0,16-12,61]	0,08

Mediana [25º, 75º percentil) por morte ou transplante de coração como resultado. CC-Dis: pacientes com sorologia positiva para T. cruzi e CC com disfunção ventricular esquerda; NT-proBNP: N-terminal do pró-hormônio do peptídeo natriurético do tipo B; NYHA: New York Heart Association. Galectina-3 baixa = <15,3 ng/mL; NT-proBNP baixo = <1278 pg/mL. *Carga parasitária por 20mL.

O valor de corte da Gal-3 (<15,3 ng/mL) pela curva ROC foi usado para dividir os indivíduos CC-Dis em dois estratos (níveis baixo e alto), assim como o valor de corte de NT-proBNP (<1278 pg/mL). A curva ROC identificou o potencial de se alcançar um evento. Embora a AUC do NT-proBNP fosse maior do que a AUC da Gal-3, não houve diferenças (p=0,22).

**Figura 2:**
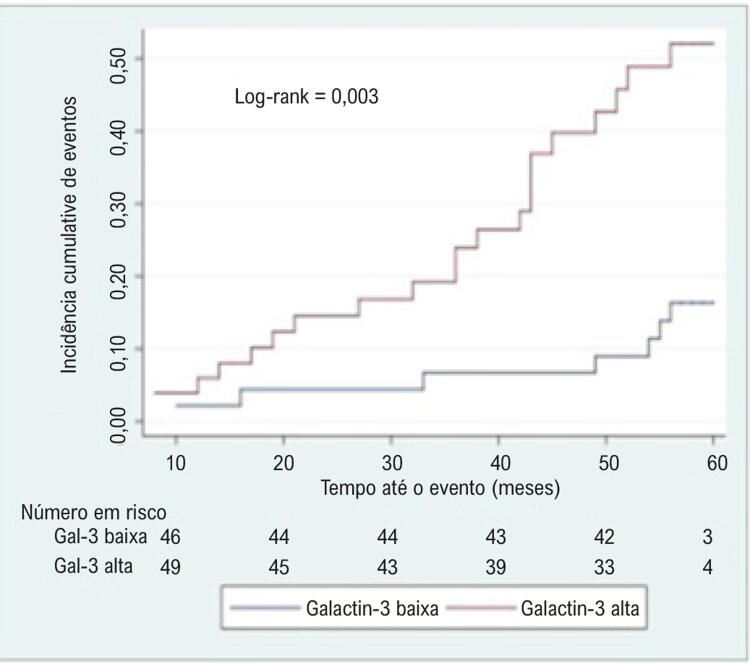
– Curvas de tempo até o evento entre os níveis de Gal-3. Curvas de tempo até o evento entre níveis estratificados de Gal-3 em CC-Dis. Valor de corte: Gal-3 <15,3 ng/mL.

Após o ajuste para sexo, idade, função renal, classe funcional da NYHA> I e FEVE, encontramos uma associação significativa de níveis mais elevados de Gal-3 com eventos subsequentes em acompanhamento de cinco anos (Tabela 3 e Figura 2). Além disso, o risco de eventos também aumentou à medida que os níveis de NT-proBNP aumentaram (Tabela 3). Resultados semelhantes foram observados quando Gal-3 e NT-proBNP (por aumento de 100 unidades) foram analisados como variáveis contínuas (Apêndice).

**Tabela 3:** – Associação entre galectina-3 e morte ou transplante cardíaco no subgrupo CC-Dis, analisada por meio de modelos de regressão Cox e curva ROC, ambos brutos e ajustados para idade, sexo, nível de creatinina sérica, classe funcional NYHA>I e LVEF, e usando Galectina -3 e NT-proBNP como variáveis categóricas

				Modelo bruto		Modelo ajustado	
	AUC	Valor de corte	Nível de corte	p	RR (IC95%)	p	RR (IC95%)
Galectina-3 (ng/mL)	0,71	15,3	Baixo				
			Alto	0,007	3,27 (1,39-7,71)	0,04	2,63 (1,00-6,90)
NT-proBNP (pg/mL)	0,80	1278	Baixo				
			Alto	<0,001	5,69 (2,41-13,42)	0,02	3,44 (1,21-9,78)

AUC: área sob a curva ROC; IC: intervalo de confiança; RR: razão de risco; FEVE: fração de ejeção do ventrículo esquerdo; NT-proBNP: N-terminal do pró-hormônio do peptídeo natriurético do tipo B; NYHA: New York Heart Association; ROC: curva característica de operação do receptor.

**Figura 3:**
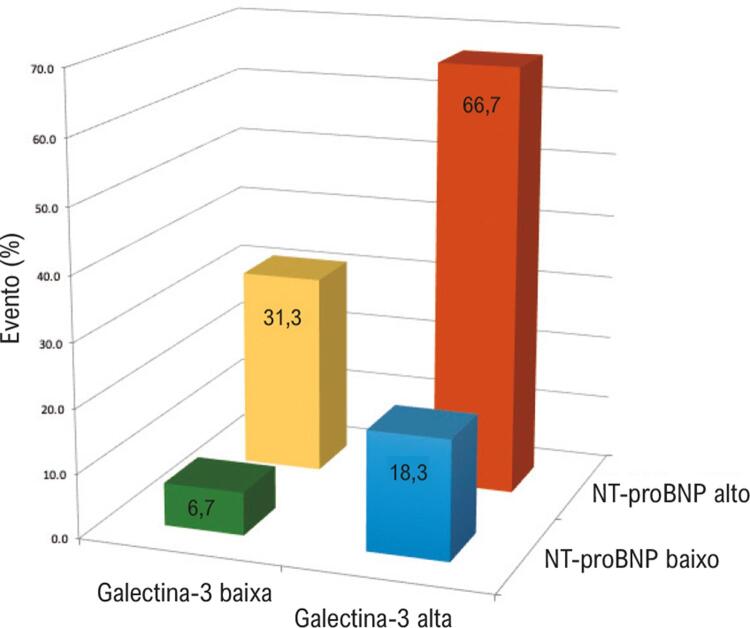
– Taxas de eventos em 5 anos em função das concentrações de Gal-3 e NT-proBNP entre pacientes com CC. A porcentagem de pacientes que experimentaram algum evento é mostrada para cada grupo. Gal-3 baixa: valores <15,3 ng/mL; Gal-3 alta: valores ≥15,3 ng/mL; NT-proBNP baixo: valores <1.278 pg/mL; NT-proBNP alto: valores ≥1.278 pg/mL.

Entre os pacientes que apresentam níveis mais elevados de Gal-3, houve diferenças nos eventos quando dicotomizados com ambos os estratos de NT-proBNP: os pacientes com níveis de NT-proBNP adicionalmente elevados tiveram probabilidade maior de evento do que os pacientes com NT-proBNP baixo. Além disso, os pacientes nos estratos de Gal-3 mais baixos, quando dicotomizados pelos níveis de NT-proBNP, foram mais propensos a qualquer evento quando o NT-proBNP era mais alto (Figura 3).

Pacientes nos estratos mais altos de Gal-3 e NT-proBNP tiveram um risco 11 a 16 vezes maior de evento em comparação com pacientes com os níveis de biomarcadores mais baixos (RR não ajustado, 16,22; IC95%: 3,71-70,83; p<0,001; RR ajustada, 11,39; IC95%: 1,97–65,76; p=0,007). Indivíduos com Gal-3 baixa e NT-proBNP baixo tiveram as taxas de eventos mais baixas.

## Discussão

Níveis aumentados de Gal-3 foi um fator significativamente associado a formas graves de DCC e preditivo de morbidade/mortalidade subsequente.

Gal-3 é um biomarcador emergente que modula vários processos fisiológicos que contribuem para a IC, inflamação e fibrose.^13-15^ A inflamação é um pré-requisito para a recuperação do tecido e formação de cicatriz,^16^ e a Gal-3 teve um papel importante como mediador em infecções parasitárias, virais^14,17^ e bacterianas revelado.^18^ Na DCC, estudos experimentais mostraram que a Gal-3 tem expressão regulada positivamente após a infecção por *T. cruzi* em células dendríticas, células B^19,20^ e macrófagos CD68+. Os macrófagos CD68+ representam 50% do infiltrado de células mononucleares em corações com CC.^21^

O *T. cruzi* e vários mecanismos imunomediados têm um envolvimento direto na CC. Estudos anteriores reportaram que a Gal-3 se liga ao 45KD, aumentando sua adesão à matriz extracelular e até mesmo sua entrada nas células. Outros estudos já demonstraram a importância da Gal-3 no processo inicial de infecção pelo *T. cruzi*, pois ela permite que o parasita se acumule na matriz extracelular antes de invadir as células hospedeiras.^22,23^

Um modelo experimental de infecção aguda por *T. cruzi *mostrou que a indução de miocardite estava associada à suprarregulação de Col I, Gal-3, IFN- e IL-13.21 A Gal-3 foi detectada principalmente em células intersticiais e em níveis mais altos em áreas fibróticas. Nas áreas de fibrose miocárdica, a intensidade da miocardite e o remodelamento extracelular significativo da matriz foram correlacionados com a presença de Col I e Gal-3. Além disso, os miofibroblastos podem induzir a fibrose, que resulta em rigidez miocárdica e disfunção cardíaca. É importante ressaltar que os miofibroblastos também são uma fonte significativa de citocinas pró-inflamatórias, incluindo TNF- e IL-1, que têm um conhecido efeito deletério no miocárdio. No entanto, não encontramos nenhuma associação significativa entre Gal-3 e marcadores inflamatórios.

Sabino et al.,^24^ compararam a detecção do DNA do T*. cruzi* com marcadores clínicos e laboratoriais conhecidos de gravidade da CC e observaram que a presença de parasitemia estava associada a marcadores de progressão da doença, como duração do intervalo QRS e QT, menor FEVE e elevação de níveis de troponina e NT-proBNP. Observou-se, também, que a detecção do DNA do *T. cruzi* foi significativamente maior nos pacientes com cardiomiopatia em comparação ao grupo Não-CC-DS; entretanto, a PCR para *T. cruzi* não se correlacionou com a Gal-3. Também não houve diferença significativa na detecção do DNA do *T. cruzi* entre pacientes com CC com e sem disfunção, nem entre pacientes com CC que experimentaram ou não eventos. Assim, em nosso estudo, o parasitismo foi um marcador de alterações típicas da cardiomiopatia no ECG, mas não da gravidade da doença ou do prognóstico clínico.

De Boer et al.,^25^ sugeriram que a Gal-3 provavelmente representa um fenótipo único de alto risco para o desenvolvimento e progressão de IC ou outras doenças cardiovasculares. Elevações crônicas da Gal-3 induzem fibrogênese ativa e podem provocar remodelação cardíaca patológica. De Boer et al.^25^ também levantaram a hipótese de que pacientes com esse fenótipo de superexpressão de Gal-3 são mais propensos a ter uma via “fibrogênica” para a remodelação cardíaca. Em nosso estudo, níveis elevados de Gal-3 foram associados à forma mais grave de cardiomiopatia, mas sem uma forte associação com parâmetros ecocardiográficos. Assim, os níveis de Gal-3 definiram uma população com doença mais grave, caracterizada por disfunção ventricular sistólica e diastólica esquerda, maior diâmetro diastólico esquerdo e direito e níveis elevados de NT-proBNP e troponina.

Echeverria et al.,^26^ examinaram o valor diagnóstico de um painel de biomarcadores para distinguir a gravidade da CC e não encontraram associações entre os níveis de sST2 e Gal-3. No entanto, a amostra era pequena e não incluiu pacientes com estádio A (*T. cruzi* positivo, mas ECG e ecocardiografia normais), o que poderia ter permitido uma análise do papel dos biomarcadores em pacientes assintomáticos, já que eles também não fornecem qualquer informação prognóstica de Gal-3.

Encontramos níveis de Gal-3 mais elevados no grupo controle em comparação ao grupo Não-CC-DS. No entanto, os valores foram menores em comparação ao grupo CC-Dis. Sabe-se, desde os estudos pioneiros de Carlos Chagas,^27^ que até 60% dos pacientes infectados não apresentam evidências que sugiram envolvimento cardiovascular ou gastrointestinal. Acredita-se que esses indivíduos tenham a forma denominada “indeterminada”, definida em nosso estudo como Não-CC-DS. Como resultado, a sobrevida nesse grupo de pacientes parece ser comparável à da população geral. Nossos resultados mostraram baixos níveis de Gal-3 neste grupo, o que suporta este conceito.

O fenótipo da galectina-3 é um fator importante no início e progressão da IC. Sabe-se que pacientes com IC com baixos níveis de Gal-3 têm progressão lenta e resultados melhores do que pacientes com IC e altos níveis de Gal-3.^25,28^Mostrou-se que a Gal-3 pode prever o desenvolvimento de mortalidade por todas as causas e IC na população geral,^28^ além de ser usada para definir e identificar pacientes com IC em risco muito baixo de mortalidade em 30 e 180 dias, e de reinternações por IC após um episódio agudo.29 Uma metanálise feita por Chen et al.30 relatou a Gal-3 sérica como fator preditor de mortalidade por todas as causas e mortalidade cardiovascular em pacientes com IC.^30^

O achado mais surpreendente de nosso estudo foi a relação entre a Gal-3 e o risco de eventos entre pacientes com CC. Como Gal-3 e NT-proBNP foram preditores independentes de eventos adversos, também mostramos que o aumento de ambos os marcadores estava associado às taxas mais altas de morte ou transplante cardíaco em pacientes com CC.

### Limitações do Estudo

Este foi um estudo realizado em um único centro, com uma amostra relativamente pequena. Além disso, fizemos apenas uma medida pontual de Gal-3 e NT-proBNP e, portanto, não avaliamos mudanças dinâmicas nesses biomarcadores ao longo do tempo. Outra limitação foi a utilização de testes não paramétricos para analisar associações entre variáveis contínuas, resultando em perda de eficiência.

## Conclusões

Níveis elevados de Gal-3 no plasma foram significativamente associados à disfunção cardíaca e gravidade da CC. Nossos resultados sugerem que uma abordagem baseada em biomarcadores para estratificação de risco em pacientes com DCC pode ajudar os médicos a identificar pacientes com maior probabilidade resultados desfavoráveis e, potencialmente, guiá-los no desenvolvimento de estratégias de tratamento para este grupo de alto risco.
