# Supplementary material for: Galectina-3 Associada a Formas Graves e Mortalidade em Longo Prazo em Pacientes com Doença de Chagas
Source: Arq Bras Cardiol. 2021 Feb 19;116(2):248–56. [Article in Portuguese] doi: 10.36660/abc.20190403 (PMC7909980; doi:10.36660/abc.20190403)
Supplement: Translation

# Translation: Galectin-3 Associated with Severe Forms and Long-term Mortality in Patients with Chagas Disease

**Authors:** Fábio Fernandes, Carlos Henrique Valente Moreira, Lea Campos Oliveira, Marcela Souza-Basqueira, Barbara Maria Ianni, Claudia di Lorenzo, Felix José Alvarez Ramires, Luciano Nastari, Edecio Cunha-Neto, Antonio L. Ribeiro, Renato Delascio Lopes, Sheila M. Keating, Ester Cerdeira Sabino, Charles Mady

**Affiliations:** 1 BrasilUniversidade de São Paulo Faculdade de Medicina Hospital das Clínicas Instituto do Coração, São Paulo, SP- Brasil; 2 BrasilInstituto de Infectologia Emilio Ribas, São Paulo, SP - Brasil; 3 BrasilUniversidade de São Paulo Faculdade de Medicina, São Paulo, SP - Brasil; 4 BrasilUniversidade Federal de Minas Gerais - Centro de Telessaúde - Hospital das Clínicas, Belo Horizonte, MG - Brasil; 5 EUADuke University Hospital, Durham, North Carolina - EUA; 6 EUABlood Systems Research Institute, San Francisco, Califórnia – EUA

**Keywords:** Chagas Disease, Chagas, Cardiomyopathy, Mortality, Galectin-3, Biomarkers, Electrocardiography/methods, Heart Failure

## Abstract

**Background:**

The histopathological characteristics of Chagas disease (ChD) are: presence of myocarditis, destruction of heart fibers, and myocardial fibrosis. Galectin-3 (Gal-3) is a biomarker involved in the mechanism of fibrosis and inflammation that may be useful for risk stratification of individuals with ChD.

**Objectives:**

We sought to evaluate whether high Gal-3 levels are associated with severe forms of Chagas cardiomyopathy (CC) and whether they are predictive of mortality.

**Methods:**

We studied anti-T. cruzi positive blood donors (BD): Non-CC-BD (187 BD without CC with normal electrocardiogram [ECG] and left ventricular ejection fraction [LVEF]); CC-Non-Dys-BD (46 BD with CC with abnormal ECG but normal LVEF); and 153 matched serum-negative controls. This cohort was composed of 97 patients with severe CC (CC-Dys). We used Kruskall-Wallis and Spearman’s correlation to test hypothesis of associations, assuming a two-tailed p<0.05 as significant.

**Results:**

The Gal-3 level was 12.3 ng/mL for Non-CC-BD, 12.0 ng/mL for CC-Non-Dys-BD, 13.8 ng/mL for controls, and 15.4 ng/mL for CC-Dys. LVEF<50 was associated with higher Gal-3 levels (p=0.0001). In our linear regression adjusted model, we found association between Gal-3 levels and echocardiogram parameters in T. cruzi-seropositive subjects. In CC-Dys patients, we found a significant association of higher Gal-3 levels (≥15.3 ng/mL) and subsequent death or heart transplantation in a 5-year follow-up (Hazard ratio – HR 3.11; 95%CI 1.21–8.04; p=0.019).

**Conclusions:**

In ChD patients, higher Gal-3 levels were significantly associated with severe forms of the disease and more long-term mortality, which means it may be a useful means to identify high-risk patients. (Arq Bras Cardiol. 2021; 116(2):248-256)

## Introduction

Chagas cardiomyopathy (CC), one of the leading causes of heart disease and death in Latin America, has a poor prognosis compared to noninflammatory cardiomyopathies.^1^

The natural history of Chagas disease (ChD) involves an acute phase, followed by the chronic phase. It is still unknown, however, which patients are more likely to progress to severe forms. Direct parasite injury, inflammation triggered by the immune system, and autonomic dysfunction are role-players in the pathogenesis of CC. When the cardiac tissue is injured, replacement fibrosis appears to be a cause of structural disorganization, geometry, and functional heart impairment.^2^

Galectin-3 (Gal-3) is secreted by activated macrophages and is involved in the fibrogenesis of heart failure (HF). This biomarker has recently been linked to development of HF and mortality. In an experimental model of ChD, Gal-3 promoted cell infiltration in the heart and fibrosis.^3,4^

The lack of a good marker of active infection or incipient CC makes the development of new treatments in this population a challenge. The use of biomarkers that can accurately predict clinical outcomes in CC would have the potential to guide therapy, by identifying patients at higher risk and who would need an earlier, more intensive, and personalized strategy.^5^

The aim of our study was to evaluate whether high Gal-3 levels are associated with severe forms of CC and whether it is a predictive factor for subsequent mortality or the need for heart transplantation.

## Methods

### Study Design

Samples were collected during the Retrovirus Epidemiology Donor Study-II (REDS-II),^6^ a retrospective cohort study in which *T. cruzi*-seropositive blood donors (BD) were identified by screening blood banks in 1996-2002 (233 from the city of Sao Paulo), in addition to 153 serum-negative control BDs (matched by year of donation, age and gender). This cohort of BD was composed of 97 previously diagnosed cases of CC from the Heart Institute (INCOR) of the Medical School of Universidade de São Paulo. From July 2008 to October 2010, recruited individuals (BD and CC patients) filled in health questionnaires and went through medical evaluations, including electrocardiogram (ECG), echocardiogram (ECHO), and phlebotomy with processing and cryopreservation of samples for subsequent batched blinded analyses of cardiac markers, polymerase chain reaction (PCR) for detection of *T. cruzi*, and other biomarkers (see below).

First, we performed a cross-sectional study in which *T. cruzi*-seropositive participants filled in a questionnaire and received a medical evaluation (laboratory, ECG, and ECHO parameters), resulting in groups stratified by CC status. Then, a longitudinal cohort study was carried out, in which 97 *T. cruzi*-seropositive patients suffering from chronic and more severe CC forms were followed up in an outpatient service from INCOR, in which time-to-event data was available.

All blood samples were collected in EDTA and serum tubes, processed for parasite detection, or submitted to spinning and divided in aliquots. All specimens were frozen in Brazil at -20°C until shipped to the REDS-II Central Laboratory (*Blood Systems Research Institute, San Francisco, CA*) on dry ice and maintained at -70°C.

Four groups were created: a control serum-negative group and three *T. cruzi* seropositive groups: BD without cardiomyopathy, presenting with normal ECG and left ventricular ejection fraction (LVEF) (Non-CC-BD); BD with CC, presenting with ECG abnormalities but normal systolic function (CC-Non-Dys-BD); and participants with CC and left ventricular dysfunction (CC-Dys). The dysfunction was defined as LVEF <50% upon ECHO.

Data regarding time-to-event analysis were available only for CC-Dys subjects. From July to September 2015, we proceeded with CC-Dys patients’ chart analysis and phone calls to monitor events and get respective dates.

The local ethics committees approved the study protocol, and all participants signed an informed consent form.

### PCR Procedures

From each participant, we collected 20 mL of EDTA-anti-coagulated blood that were immediately mixed with an equal volume of 6 M guanidine hydrochloride -0.2M EDTA solution. The target-capture real-time PCR assay used in this study was developed based on the PCR method described by Virreira et al.,^7^ targeting the kinetoplast *T. cruzi* mitochondrial minicircle DNA. The DNA extraction was improved with a target-capture step using magnetic beads coated with a *T. cruzi*-specific 20-mer capture oligonucleotide.

### Cardiac Measurements

Resting 12-lead ECGs were recorded (*General Electric MAC 1200 electrocardiograph; GE Healthcare, Waukesha, WI*).^8,9^

Echocardiograms were performed using a Sequoia^TM^ 512 ultrasound machine (*Acuson, Mountain View, C*A), per guidelines by the American Society of Echocardiography.^10,11^ Studies were recorded in digital format, and all measurements were made on digital loops using a *Digisonics* offline analysis station (*version 3.2 software; Digisonics, Houston, TX*) at the Cardiovascular Branch, Echocardiography Laboratory, of the National Heart, Lung, and Blood Institute (*Bethesda, MD*).^12^

### Soluble Biomarkers

Biomarkers associated with *T. cruzi *infection have been previously described.^12^ Blinded plasma samples were tested using Milliplex kits (*Millipore*) with antibody-coated beads for detection of tumor necrosis factor alpha (TNF-α), interleukin (IL) 6 (IL-6), IL-8, IL-10, and interferon gamma (IFNγ). Standard curves and samples were tested in duplicate. Results were acquired on a Labscan 200 analyzer (Luminex) using the Bio-Plex manager software v6.1 (Bio-Rad). IFNγ was predominantly below the threshold of detection (57%).

Concentrations of N-terminal pro B-type natriuretic peptides (NT-proBNPs) and troponin were measured using U.S. Food and Drug Administration-cleared assays on the VITROS System (*Ortho Clinical Diagnostics, Raritan, NJ*).

Plasma Gal-3 levels were determined by an enzyme-linked fluorescence assay and measured on a BioMerieux Vidas 30 system (*BioMerieux, Marcy l’Etoile, Lyon, France*), following the manufacturer’s recommendations.

### Statistical Analyses

Normality was tested with the Shapiro-Wilk test. Continuous non-normally distributed variables were: age, Gal-3 levels, ejection fraction, and other cardiac and inflammatory biomarkers, being expressed as median and interquartile range. Differences between groups as to variables were compared using the Kruskal-Wallis test, while chi-squared or Fisher’s test and logistic regression were used to assess variable type and distribution. Use of ranks in one-criterion variance analysis and post-hoc analysis was made with Dunn’s test to evaluate median-value differences between the groups with Bonferroni adjustment for multiple comparisons. For analysis of correlations, the Spearman’s correlation was used, reporting p values.

Receiver operating characteristic (ROC) curves were performed for Gal-3 and NT-proBNP to optimize the definition of the cutoff points that would best discriminates the event at follow-up, and an area under the curve (AUC) were identified. Both curves were compared with DeLong and chi-squared tests.

For a time-to-event analysis, the CC-Dys group was divided in two profiles regarding Gal-3 and NT-proBNP cutoff values: low Gal-3 or NT-proBNP (≤ cutoff) and high Gal-3 or NT-proBNP (≥ cutoff). Analysis of incidence of cumulative events across Gal-3 and NT-proBNP strata and the additive value of Gal-3 relative to NT-proBNP was made by a Kaplan-Meier-like method followed by log-rank test.

Bivariate and multivariate Cox proportional-hazards regression models were constructed to evaluate the association of Gal-3 and NT-proBNP values (below *versus *above or equal to the cutoff value) with incident events. Models were adjusted for sex, age, serum creatinine, New York Heart Association (NYHA) classification and LVEF; 95% confidence intervals (CI) were used to depict the association of each marker and the events in the final fitted Cox proportional-hazards model. Sensitivity was analyzed using Gal-3 and NT-proBNP as continuous variables. A two-tailed p<0.05 was considered as significant.

All graphs and statistical analyses were made in the software Stata (*version 13.0, Stata Corp., College Station, TX*).

## Results

Of the original 570 participants in REDS-II, 483 had samples available for Gal-3 testing; 153 were anti-*T. cruzi* serum-negative BD, and the remainder were sorted into three groups: 187 Non-CC-BD presenting with normal ECG and LVEF; 46 CC-non-Dys; and 97 CC-Dys (Figure 1).

**Figure 1:** – Flowchart of inclusion.

*CC: Chagas cardiomyopathy; Non-CC-BD: blood donors with positive T. cruzi serology and without cardiomyopathy, presenting normal electrocardiogram (ECG) and left ventricular ejection fraction (LVEF); CC-Non-Dys-BD: blood donors with positive T. cruzi serology and presenting ECG abnormalities and normal LVEF at rest; CC-Dys: patients with positive T. cruzi serology and CC, with left ventricular dysfunction.*

### Patient Clinical and Biomarker Characteristics

Demographic and clinical characteristics are described in Table 1. Gal-3 levels were greater in patients from the CC-Dys group, than other clinical groups. Higher Gal-3 levels were also seen in controls when compared with Non-CC-BD. We did not observe significant differences in Gal-3 levels between Non-CC-BD and CC-non-Dys-BD, or between CC-non-Dys-BD and controls. Inflammatory markers (TNF-α, IL-6, IL-8, IL-10), as well as biomarkers associated with, cardiac dysfunction or damage (NT-proBNP and troponin) were elevated in CC-Dys patients compared to other groups (Table 1).

**Table 1:** – Clinical, laboratory, and echocardiographic findings

	Non-CC-BD	CC-Non-Dys-BD	CC-Dys	Controls	
	n=187 (38.6%)	n=46 (9.5%)	n=97 (20.2%)	n=153 (31.6%)	p-value
Age (y)	49 [41-58]	50 [44-59]	48.5 [43-54]	48 [42-55]	0.20
Male, n (%)	110 (58.8)	29 (63)	59 (60.2)	89 (58.2)	0.39
Ejection fraction (%)	63 [60-65]	60 [55-65]	30 [20-40]	64 [60-65]	<0.001*
**Cardiac biomarkers**					
Galectin-3 (ng/mL)	12.3 [10-15.4]	12.0 [9.5-14.9]	15.4 [11.8-19.8]	13.8 [11.2-16.2]	<0.001^†^
NT-proBNP (pg/mL)	40.6 [23.6-66.8]	59 [35.0-109.1]	748 [379.20-2223.41]	27.5 [19.3-48.1]	<0.001^‡^
Troponin	0.012 [0.01-0.012]	0.012 [0.012-0.015]	0.021 [0.12-0.03]	0.012 [0.012-0.012]	<0.001§
**Inflammatory markers**					
TNF-α	2.94 [1.64-4.59]	3.02 [1.25-4.69]	3.65 [2.57-5.52]	2.84 [1.62-3.91]	0.002^#^
IL-6	0.69 [0.32-1.63]	0.77 [0.32-1.8]	1.60 [0.64-3.13]	1.14 [0.32-1.7]	<0.001 **
IL-8	1.61 [0.95-2.79]	1.53 [0.99-2.5]	2.23 [1.38-3.2]	1.44 [0.95-2.54]	0.003^††^
IL-10	1.28 [0.32-4]	2.02 [0.32-4.11]	4.37 [1.62-8.06]	1.22 [0.32-3.35]	<0.001^‡‡^
IFN-γ	0.32 [0.32-0.64]	0.32 [0.32-0.84]	0.32 [0.32-1.07]	0.32 [0.32-0.39]	0.06
**Parasite load**					
**Parasite estimate per 20mL**	**0.05 [0-2.5]**	**0.68 [0.03-5.47]**	**1.77 [0.16-5]**	-	**<0.001^§§^**

p-values were reported for Kruskal-Wallis and Dunn post-hoc hypothesis testing. Median [interquartile range] reported for all biomarkers tested. Non-CC-BD, blood donors without Chagas cardiomyopathy; CC-Non-Dys-BD, blood donors with Chagas cardiomyopathy; CC-Dys, Chagas cardiomyopathy patients with cardiac dysfunction; NT-proBNP, N-terminal pro B-type natriuretic peptide; TNF, tumor necrosis factor; IL, interleukin; IFN, interferon. *Statistically significant difference in ejection fraction levels between Non-CC BD/CC-Dys (p<0.001); CC-Non-Dys-BD/CC-Dys (p<0.001); CC-Non-Dys-BD/controls (p=0.042); CC-Dys/controls (p<0.001). †Statistically significant difference in Gal-3 levels between Non-CC-BD/CC-Dys (p<0.001); Non-CC-BD/controls (p=0.010); CC-Non-Dys-BD/CC-Dys (p<0.001); CC-Dys/controls (p=0.028). ‡Statistically significant difference in NT-proBNP levels between Non-CC-BD/CC-Dys (p<0.001); Non-CC-BD/controls (p=0.004); CC-Non-Dys-BD/CC-Dys (p<0.001); CC-Non-Dys-BD/controls (p<0.001); CC-Dys/controls (p<0.001). §Statistically significant difference in troponin levels between Non-CC-BD/CC-Dys (p=0.024); Non-CC-BD/CC-controls (p<0.001); CC-Non-Dys-BD/CC-Dys (p<0.001); CC-Non-Dys-BD/controls (<0.001); CC-Dys/controls (p<0.001). #Statistically significant difference in TNF-α levels between Non-CC-BD/CC-Dys (p=0.019); CC-Dys/controls (p<0.001). **Statistically significant difference in IL-6 levels between Non-CC-BD/CC-Dys (p<0.001); CC-Non-Dys-BD/CC-Dys (p=0.032); CC-Dys/controls (p=0.004). ††Statistically significant difference in IL-8 levels between Non-CC-BD/CC-Dys (p=0.016); CC-Non-Dys-BD/CC-Dys (p=0.039); CC-Dys/controls (p=0.001). ‡‡Statistically significant difference in IL-10 levels between Non-CC-BD/CC-Dys (p<0.001); CC-Non-Dys-BD/CC-Dys (p=0.001); CC-Dys/controls (p<0.001). §§Statistically significant difference in parasite estimate per 20mL between Non-CC-BD/CC-Dys (p=0.011); Non-CC-BD/controls (p<0.001).

Positive *T. cruzi *PCR indicated a statistically significant difference between Non-CC-BD and CC groups: CC-Non-Dys-BD (p=0.010) and CC-Dys. By contrast, no difference in parasitemia was observed when comparing CC-Non-Dys-BD and CC-Dys (Table 1). However, we did not finda

significant difference in CC-Dys patients between *T. cruzi* PCR and event occurrence. No significant association between *T. cruzi* PCR and Ga*l-3 was found.*

Among the T. cruzi infected subjects, Spearman’s correlation was applied to assess the relationship between Gal-3 and cardiac biomarkers, inflammatory mediators, and parasite load. There was a weak correlation for TNF- (r_s_=0.25, p<0.001) and IL-8 (r_s_=0.22, p<0.001). By contrast, no association between Gal-3 and Troponin, NT-proBNP, IL-6, IL-10, IFN-, or with parasite load was found.

Echocardiography was carried out, and we used Spearman’s correlation, to verify any relationship between Gal-3 levels and echocardiographic parameters among T. cruzi infected patients. No moderate or strong statistically significant correlation was found between Gal-3 levels and left ventricular end-diastolic diameter (LVEDD) (rs=0.09, p=0.07), left ventricular end-systolic dimension (LVESD) (rs=0.11, p=0.03), LVEF (r_s_=-0.16, p=0.001), left atrial diameter (r_s_=0.11, p=0.02), left atrial volume indexed to body surface area (r_s_=0.09, p=0.18), right atrial area (r_s_=0.032, p=0.53) and septal E/e’ ratio (r_s_=0.135, p=0.009).

### Survival and Risk Analysis

Time-to-event data were available for 97 patients, with mean follow-up of 51.2 ± 10.8 months and median of 58 months (range: 8 to 60 months). Events were observed in 28 patients (29%), and were due to three (10.8%) heart transplantations and 25 (89.2%) deaths by all causes. Among event-experienced patients, median concentrations of Gal-3 and NT-pro-BNP were significantly higher, while the ejection fraction was significantly lower. Age, sex, NYHA class >I, ejection fraction on echocardiography, and laboratory data of event-experienced patients are compared in Table 2.

**Table 2:** – Demographic and laboratory information of CC-Dys patients according to death or heart transplantation

	No events	Events	p-value
	(n=69)	(n=28)	
Age (y)	49 [42-54]	47.5 [44.5-52]	0.96
Male, n (%)	42 (61%)	16 (57%)	0.73
Creatinine (mg/dL)	1.01 [0.85-1.14]	1.14 [0.87-1.23]	0.06
Ejection fraction (%)	35 [25-40]	20 [20-30]	0.001
NYHA >1	29	20	0.009
Galectin-3 (ng/mL)	14.4 [10.9-19.1]	18.5 [14.7-23.4]	0.005
Low	39	7	
High	30	21	
NT-proBNP (pg/mL)	542 [281-1337]	2643 [1047-4771]	<0.001
Low	50	7	
High	19	21	
Parasite estimate*	1.77 [0.19-4.2]	1.25 [0.16-12.61]	0.08

Median [25^th^, 75^th^ percentile) by death or heart transplantation as an outcome. CC-Dys: patients with positive T. cruzi serology and CC with left ventricular dysfunction; NT-proBNP: N-terminal pro B-type natriuretic peptide; NYHA: New York Heart Association. Low Galectin-3 = <15.3 ng/mL; Low NT-proBNP = <1278 pg/mL. *Parasite load per 20mL.

Gal-3 cutoff point (<15.3 ng/mL) by ROC curve was used to divide CC-Dys subjects into two strata (low and high levels), as was the NT-proBNP cutoff point (<1278 pg/mL). ROC identified the potential to reach an event. Although the AUC of NT-proBNP was larger than the Gal-3 AUC, there were no differences (p=0.22).

After adjusting for sex, age, renal function, NYHA functional class >I, and LVEF, we found a significant association of higher levels of Gal-3 with subsequent events in a 5-year follow-up (Table 3 and Figure 2). Complementarily, the risk of events also increased as the levels of NT-proBNP climbed (Table 3). Similar results were seen when Gal-3 and NT-proBNP (by 100-unit increase) were analyzed as continuous variables (Appendix).

**Table 3:** – Association between galectin-3 and death or heart transplantation in the CC-Dys subgroup using ROC and Cox regression models, both crude and adjusted for age, sex, serum creatinine level, NYHA functional class >I and LVEF, and using Galectin-3 and NT-proBNP as categorical variables

				Crude Model		Adjusted Model	
	AUC	Cutoff	Cutoff Level	p	HR (95%CI)	p	HR (95%CI)
Galectin-3 (ng/mL)	0.71	15.3	Low				
			High	0.007	3.27 (1.39-7.71)	0.04	2.63 (1.00-6.90)
NT-proBNP (pg/mL)	0.80	1278	Low				
			High	<0.001	5.69 (2.41-13.42)	0.02	3.44 (1.21-9.78)

AUC: area under the curve; CI: confidence interval; HR: hazard ratio; LVEF: left ventricular ejection fraction; NT-proBNP: N-terminal pro B-type natriuretic peptide; NYHA: New York Heart Association; ROC: receiver operating characteristic

**Figure 2:** – Time-to-event curves between Gal-3 levels. Time-to-event curves between stratified Gal-3 levels results in CC-Dys. Cutoff level: Gal-3 <15.3 ng/mL.

Among patients presenting higher Gal-3 levels, we found differences in events when dichotomized with both NT-proBNP strata: patients with additionally high NT-proBNP levels were more likely to experience any event than patients with low NT-proBNP. Moreover, patients in the lower Gal-3 strata, when dichotomized with NT-proBNP levels, were more likely to present any event when NT-proBNP was higher (Figure 3).

**Figure 3:** – Event frequency at 5-year as a function of Gal-3 and NT-proBNP concentrations among CC patients. The percentage of patients who experienced any event is shown for each group. Low Gal-3: values <15.3 ng/mL; high Gal-3: values ≥15.3 ng/mL; low NT-proBNP: values <1,278 pg/mL; high NT-proBNP: values ≥1,278 pg/mL.

Patients in the higher strata of both Gal-3 and NT-proBNP levels had 11 to 16-fold increased risk of event compared with those with the lowest biomarker levels (unadjusted HR, 16.22; 95%CI: 3.71–70.83; p<0.001; adjusted HR, 11.39; 95%CI: 1.97–65.76; p=0.007). Subjects with low Gal-3 and low NT-proBNP had the lowest event rates.

## Discussion

Increased Gal-3 was significantly associated with severe forms of ChD and predictive of subsequent morbidity/mortality.

Gal-3 is an emerging biomarker and modulates several physiological processes that contribute to HF, inflammation, and fibrosis.^13-15^ Inflammation is a prerequisite for tissue healing and scar formation,^16^ and Gal-3 has been shown to play a major role as a mediator in parasitic, viral,^14,17^ and bacterial infection.^18^ In ChD, experimental studies have shown that Gal-3 has upregulated expression following *T. cruzi* infection in dendritic cells, B cells,^19,20^ and CD68+ macrophages. Significantly, CD68+ macrophages represent 50% of the mononuclear cell infiltrate in hearts with CC.^21^

*T. cruzi* and several immune-mediated mechanisms have a direct involvement in CC. Previous studies have reported that Gal-3 binds to 45KD, enhancing its adhesion to the extracellular matrix and even its entry into cells. Other studies have shown the importance of Gal-3 in the early process of *T. cruzi* infection, as it allows parasites to accumulate in the extracellular matrix before invading host cells.^22,23^

An experimental model of acute *T. cruzi* infection showed that induction of myocarditis was associated with the upregulation of Col I, Gal-3, IFN-, and IL-13.^21^ Gal-3 was primarily detected in interstitial cells and was higher in fibrotic areas. In myocardial areas of fibrosis, the intensity of myocarditis and significant matrix extracellular remodeling was correlated with the presence of Col I and Gal-3. In addition, myofibroblasts can induce fibrosis, which results in myocardial stiffness and cardiac dysfunction. Importantly, myofibroblasts are also a significant source of proinflammatory cytokines, including TNF-α and IL-1, which have a known deleterious effect on the myocardium. However, we did not find any significant association between Gal-3 and inflammatory markers.

Sabino et al.^24^ compared the detection of *T. cruzi* DNA with known clinical and laboratory markers of CC severity and observed that the presence of parasitemia was associated with markers of disease progression, such as QRS and QT interval duration, lower LVEF, and elevated troponin and NT-proBNP levels. It was also observed that detection of *T. cruzi* DNA was significantly higher in patients with cardiomyopathy as compared to Non-CC-BD group; however, *T. cruzi* PCR did not correlate with Gal-3. Moreover, there was no significant difference in the detection of *T. cruzi* DNA between CC patients with and without dysfunction, nor between CC patients who did or did not experience events. Thus, in our study, parasitism was a marker of typical ECG changes in cardiomyopathy, but not of disease severity or clinical prognosis.

De Boer et al.^25^ suggested that Gal-3 likely represents a unique phenotype at high risk for the development and progression of HF or other cardiovascular diseases. Chronic elevations in Gal-3 induce active fibrogenesis and may provoke pathological cardiac remodeling. De Boer et al.^25^ also hypothesized that patients with this phenotype of Gal-3 overexpression are more likely to have a “fibrogenic” pathway for cardiac remodeling. In our study, high levels of Gal-3 were associated with the most severe form of cardiomyopathy, but without a strong association with echocardiographic parameters. So, Gal-3 levels defined a population with more severe disease, characterized by left systolic and diastolic ventricular dysfunction, higher left and right diastolic diameter, and elevated of NT-proBNP and troponin levels.

Echeverria et al.^26^ examined the diagnostic value of a panel of biomarkers to distinguish the severity of CC and found no associations between sST2 and Gal-3 levels. However, the sample size was small and did not include patients with stage A (positive *T. cruzi*, but normal ECG and echocardiography), which could have allowed the examination of the role played by the biomarkers in asymptomatic patients. They also do not provide any prognostic information of Gal-3.

We found higher Gal-3 levels in the control group compared to the Non-CC-BD group. However, the values were lower compared to the CC-Dys group. It is known, since Carlos Chagas’ pioneering studies,^27^ that up to 60% of infected patients have no evidence suggesting cardiovascular or gastrointestinal involvement. These individuals are thought to have the so-called indeterminate form, defined as Non-CC-BD in our study. As a result, survival in this group of patients appears to be comparable to the general population. Our results showed low Gal-3 levels in this group, which supports this concept.

Galectin-3 phenotype is an important factor in the onset and progression of HF. It is known that HF patients with low Gal-3 levels have slow progression and better outcomes than patients with HF and high Gal-3 levels.^25,28^ Gal-3 was shown to predict the development of all-cause mortality and HF in the general population^28^ and can be used to define and identify patients with HF at very low risk for 30-day and 180-day mortality, and HF rehospitalizations after an episode of acute HF.^29^ A meta-analysis by Chen et al.^30^ reported the value of serum Gal-3 as a predictor factor of all-cause mortality and cardiovascular mortality in HF patients.^30^

Our study’s most striking finding was the relationship between Gal-3 and the risk of events among patients with CC. Because both Gal-3 and NT-proBNP were independent predictors of adverse events, we also showed that the increase of both markers was associated with the highest rates of death or heart transplantation in patients with CC.

### Study Limitations

This was a single-center study with a relatively small sample. In addition, we had only a single-time point measure of Gal-3 and NT-proBNP and, therefore, did not assess dynamic changes in these biomarkers over time. Another limitation was the use of non-parametric tests to analyze associations between continuous variables, resulting in loss of efficiency.

## Conclusions

High plasma Gal-3 levels were significantly associated with cardiac dysfunction and CC severity. Our findings suggest that a biomarker-based approach for risk stratification in ChD patients might help physicians identify patients who are more likely to have worse outcomes and potentially guide the development of treatment strategies for this high-risk group.
